# Racial and socioeconomic disparities in survival improvement of eight cancers

**DOI:** 10.1038/s44276-024-00044-y

**Published:** 2024-03-11

**Authors:** Vikram Shaw, Baoyi Zhang, Mabel Tang, William Peng, Christopher Amos, Chao Cheng

**Affiliations:** 1 Institute for Clinical and Translational Research, Baylor College of Medicine, Houston, TX 77030 USA; 2 Department of BioSciences, Biochemistry, and Cell Biology, Rice University, Houston, TX 77005 USA; 3 University of Houston, Houston, TX 77004 USA; 4 Department of Medicine, Section of Epidemiology and Population Sciences, Baylor College of Medicine, Houston, TX 77030 USA; 5 Dan L Duncan Comprehensive Cancer Center, Baylor College of Medicine, Houston, TX 77030 USA

## Abstract

**Background:**

Many studies have characterized racial differences in cancer outcomes, demonstrating that black and Hispanic patients have lower cancer-specific survival compared to white patients. However, to our knowledge, a gap in the literature exists regarding racial, socioeconomic, age, and sex-related differences in survival improvement in cancer.

**Methods:**

We perform a population-based cohort study of 1,875,281 patients with eight common cancer sites from the Surveillance, Epidemiology, and End Results (SEER) database.

**Results:**

The longitudinal data demonstrates that while overall cancer-free survival has improved from 2004 to 2018, certain groups have seen lower rates of improvement. Black patients have the lowest cancer-specific survival (CSS) in breast, prostate, ovarian, colon, liver, lung, and pancreatic cancers. However, from 2009 to 2018, black patients have seen the greatest survival improvement in breast, ovarian, colorectal, liver, lung, and pancreatic cancer, though CSS for black patients still lags behind other groups. Strikingly, however, in breast and prostate cancer, black patient CSS from 2014 to 2018 remains lower than white patient CSS from 2004 to 2008 after controlling for income, age, and stage.

**Conclusions:**

While the racial disparity gap is closing in some forms of cancer, future research should focus on identifying factors causing disparate outcomes to help reduce cancer-related disparities.

## Introduction

Cancer is the second leading cause of human deaths in the US and many other countries, causing around 10 million deaths worldwide per year [1, 2] Different cancer sites are typically associated with different survival, with favorable survival time in breast and prostate cancer and unfavorable survival time in lung and pancreatic cancer [2]. During the past two decades, cancer treatment options have greatly advanced, resulting in significantly improved survival in recent years [3] Notably, however, survival improvements vary across different cancer sites.

In the same cancer type, the survival of different patient subsets can also vary dramatically depending on race, age, sex, and socioeconomic status, a phenomenon known as cancer disparity. Studies have reported worse survival for black patients compared to white patients in breast [4] and prostate [5] cancer and better survival for females compared to males in lung cancer [6, 7]. Additionally, young age at diagnosis and high socioeconomic status are commonly associated with favorable prognosis in nearly all cancer sites [8, 9].

Although disparities in survival have been extensively investigated across cancer sites, disparities in survival improvements remain poorly studied. Investigating such disparities is important as it may help us understand the underlying causes and forecast the future trend of survival disparities. Based on our findings, strategies such as optimizing treatment options and reallocating healthcare resources can be used to reduce survival disparity problems. A previous study revealed disparities in survival improvements for patients diagnosed between 1990 and 2009, with significant survival disparities between age groups and narrowed survival disparities between racial groups, except for ovarian cancer [10]. In the past decade, the survival of almost all cancer sites has improved substantially [3, 11, 12] thanks to the availability of new treatment options, especially the wide application of immunotherapies [13, 14]. Our previous work has also demonstrated survival improvement in prostate cancer, but the results have not yet been expanded and compared across additional cancer sites [15]. As such, it is critical to revisit the racial disparity issue in the context of survival improvement across different cancer sites. In this study, we used population-based cancer registry data collected by the Surveillance, Epidemiology, and End Results (SEER) database to evaluate survival improvements in different patient subsets according to race, age, socioeconomic status and sex in eight cancer sites from 2004–2018.

## Methods

### SEER database

We analyzed SEER 18 registries Incidence-Based Mortality data for cancer patients diagnosed during 2004–2018. Eight cancers of the most common cancer sites were selected for this study (Supplementary Table 1). To ensure high data quality, we applied the following criteria to select patients: (1) the “Type of Reporting Source” is “Hospital inpatient/outpatient or clinic”, (2) the patient is diagnosed with only one primary cancer as indicated by “One primary only” in the variable “Sequence number” provided by SEER, (3) age of diagnosis is between 40 and 85.

The overall and cancer-specific survival (CSS) information was determined based on the variables “Vital status recode (study cutoff used)”, “SEER cause-specific death classification” and “Survival months”. The race information was determined by the variable “Race and origin recode (NHW, NHB, NHAIAN, NHAPI, Hispanic)”, from which “Non-Hispanic White”, “Non-Hispanic Black”, “Non-Hispanic Asian or Pacific Islander”, and “Hispanic (All Races)” were categorized as White, Black, Asian, and “Hispanic”. Of note, Hispanic is an ethnic category and not considered a racial category. Except for breast cancer, cancer stages were determined based on “Derived AJCC Stage Group, 6th ed (2004–2015)”, “Derived SEER Cmb Stg Grp (2016–2017)”, and “Derived EOD 2018 Stage Group (2018+)” for patients diagnosed during 2004–2015, 2016–2017, and 2018, respectively. For breast cancer, cancer stages were determined based on “Breast - Adjusted AJCC 6th Stage (1988–2015)”, “Derived SEER Cmb Stg Grp (2016–2017)”, and “Derived EOD 2018 Stage Group (2018+)” for patients diagnosed during 2004–2015, 2016–2017, and 2018. The age, sex and annual income information were determined based on “Age recode with single ages and 85+”, “Sex” and “Median household income inflation adj to 2019”, respectively. Of note, the prostate cancer cohort in our previous study [15] was 534,076 and 506,717 in the present study.

### Statistical analysis

We performed survival analysis focusing on CSS with R package “survival”. Multivariable Cox regression was used to estimate survival disparities in different patient subgroups according to race, age, income and sex. Specifically, we stratified patients into three age groups: 40–55 (Younger), 56–70 (Middle), and 71–85 (Older) groups; and three income groups: “< $60,000” (Low), “$60,000–$74,999” (Intermediate), and “>$75,000” (High). Income groups were selected to straddle the median US income of ~$70,000. To investigate racial, age, socioeconomic and sexual disparities, we used white race, younger age, low income and female sexes as reference groups, respectively.

To investigate survival improvement disparities, we grouped patients into three 5-year bins according to their year of diagnosis: 2004–2008, 2009–2013, 2014–2018; and used 2004–2008 as the reference group. We used multivariable Cox regression to calculate hazard ratios (HRs) and 95% CIs for each 5-year survival increment in different patient subgroups according to race, age, income and sex. *P* values less than 0.05 were considered as significant.

## Results

### Cancer-specific survival patterns by race, age, income, and sex

We investigated CSS after 1, 3, and 5 years for each cancer type, stratified by race, age, income, and sex (Supplementary Table 2). White patients have the highest CSS in breast, prostate, and rectal cancers, while Asian patients have the highest CSS in ovarian, liver, lung, and pancreatic cancers. Black and Hispanic patients have the lowest CSS in breast and prostate cancers, and black patients also demonstrate the lowest CSS in ovarian, colon, liver, lung, and pancreatic cancers.

As expected, the oldest age group, age 71–85 at diagnosis, has the worst CSS in all cancer sites, while the youngest group, age 40–55 at diagnosis, has the highest CSS in all cancer sites. High-income patients have the highest CSS in ovarian, liver, lung, and pancreatic cancers but the lowest CSS in breast and prostate cancers. Finally, female patients overall have higher CSS in all cancers, except for rectal cancer, when compared to male patients.

### Racial disparities in cancer-specific survival for a wide array of cancers

**Fig. 1:**
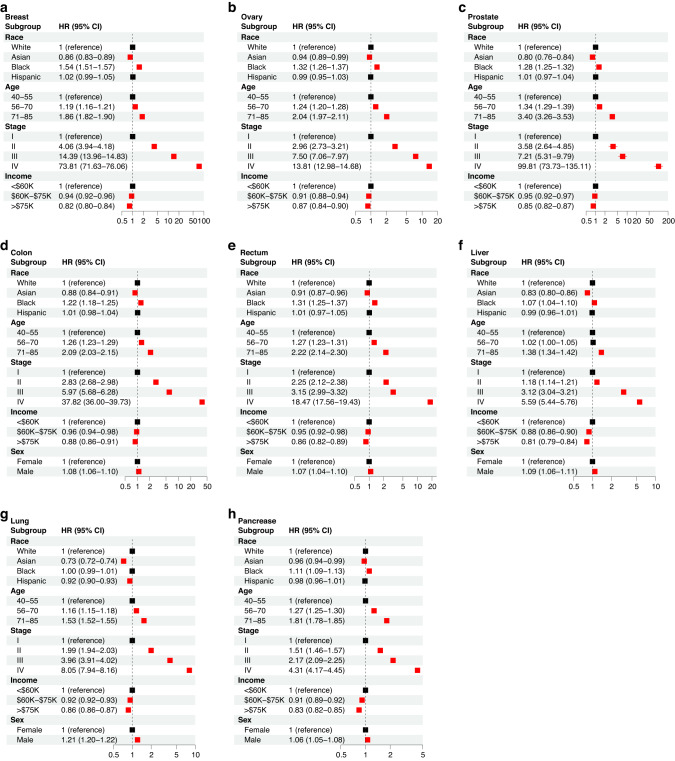
Multivariable Cox regression analysis to determine the effects of race, age at diagnosis, stage, income, and sex on CSS for each cancer type. The hazard ratio (HR) and 95% confidence interval (CI) is demonstrated for breast (**a**), ovary (**b**), prostate (**c**), colon (**d**), rectum (**e**), liver (**f**), lung (**g**), and pancreatic (**h**) cancer. The reference group for each comparison is denoted with an HR of 1 and the “reference” label.

We performed stratified cross-sectional analyses to determine the effects of race, age at diagnosis, socioeconomic status, sex, and cancer stage for each cancer type (Fig. 1). Our analyses indicated that for all cancer sites, except lung cancer, black patients demonstrate a significantly lower CSS when compared to white patients, with the highest disparity observed in breast cancer. Asian patients have a significantly longer CSS in all cancer sites. For lung cancer in particular, Asian patients have the highest survival advantage (adjusted HR = 0.73, CI = [0.72, 0.74]). Hispanic patients have significantly higher CSS in lung cancer (adjusted HR = 0.92, CI = [0.90–0.93]), but differences in CSS are not significant in the other cancer sites. Additionally, a cross-sectional age disparity was observed in all cancer sites. In breast, ovarian, prostate, colon, rectal, lung, and pancreatic cancer, the 56–70 and 71–85 age groups show an increased risk of cancer-specific death when compared to the younger group (40–55).

Significant socioeconomic disparities with consistent patterns were observed in all cancer sites. With the increase of adjusted annual household income, patients show a lower risk of CSS for all cancer sites. Finally, in all cancer sites affecting both men and women, male patients have a significantly higher HR when compared to female patients. Furthermore, as expected, in all cancer sites, diagnosis with more advanced stages is associated with lower CSS. In most cancers, Stage IV shows a higher HR than the other three stages.

### Overall cancer-free survival has improved from 2004 to 2018, though disparities remain

**Fig. 2:**
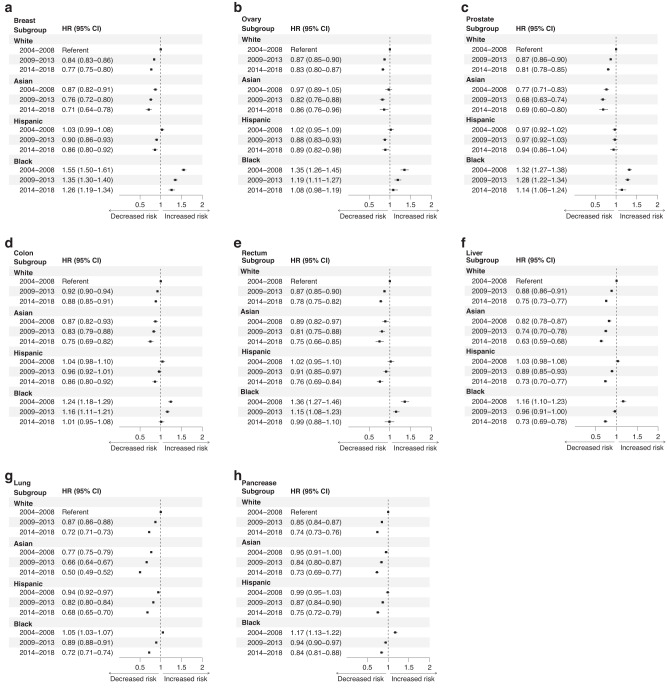
Multivariable Cox regression analysis to examine changes in CSS by race between 2004 and 2018. The hazard ratio (HR) and 95% confidence interval (CI) is demonstrated for breast (**a**), ovary (**b**), prostate (**c**), colon (**d**), rectum (**e**), liver (**f**), lung (**g**), and pancreatic (**h**) cancer. The referent group for each cancer type is white patients from 2004 to 2008.

Next, we examined the improvement in survival for by race for each 4-year period between 2004 and 2018. Our results indicate that all races show an improvement in survival for most cancer sites between 2009–2013 and 2014–2018 when compared to 2004–2008 (Fig. 2). White patients experienced a significant improvement in CSS in all cancer sites in each time period. A similar improvement was seen in Hispanic patients, with a significant improvement in survival in all cancers and time periods, except for prostate cancer survival. Asian patients also demonstrated significant improvements in survival for most cancer sites, except for colon cancer. Black patients experienced the greatest improvement in CSS comparing 2014–2018 with 2004–2008 in breast, ovarian, colon, rectal, liver, lung, and pancreatic cancer. However, in breast, ovarian, and prostate cancer, black patients from 2014 to 2018 still demonstrate lower survival than white patients from 2004 to 2008, suggesting a persistent disparity in these three cancer sites.

### Younger patients demonstrate improved survival across many cancer sites

**Fig. 3:**
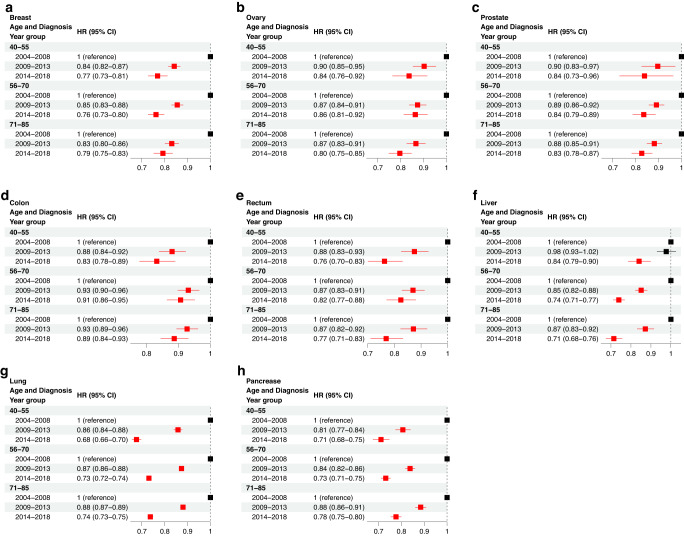
Multivariable Cox regression analysis to examine changes in CSS due to age at diagnosis between 2004 and 2018. The hazard ratio (HR) and 95% confidence interval (CI) is demonstrated for breast (**a**), ovary (**b**), prostate (**c**), colon (**d**), rectum (**e**), liver (**f**), lung (**g**), and pancreatic (**h**) cancer. The reference group for each comparison is denoted with an HR of 1 and the “reference” label.

We next examined changes in CSS due to age at diagnosis between 2004 and 2018. Notably, significant improvements in survival were achieved for all age groups in all cancer sites (Fig. 3). In colon, lung, and pancreatic cancers specifically, the youngest group achieved the greatest improvement in survival while in ovarian and liver cancers, the older groups achieved the greatest survival improvement. It is notable that for colon and rectal cancer, the middle age group 56–70 had the lowest improvement when compared to the younger and older age groups.

### Higher income patients demonstrate improved survival across many cancer sites

**Fig. 4:**
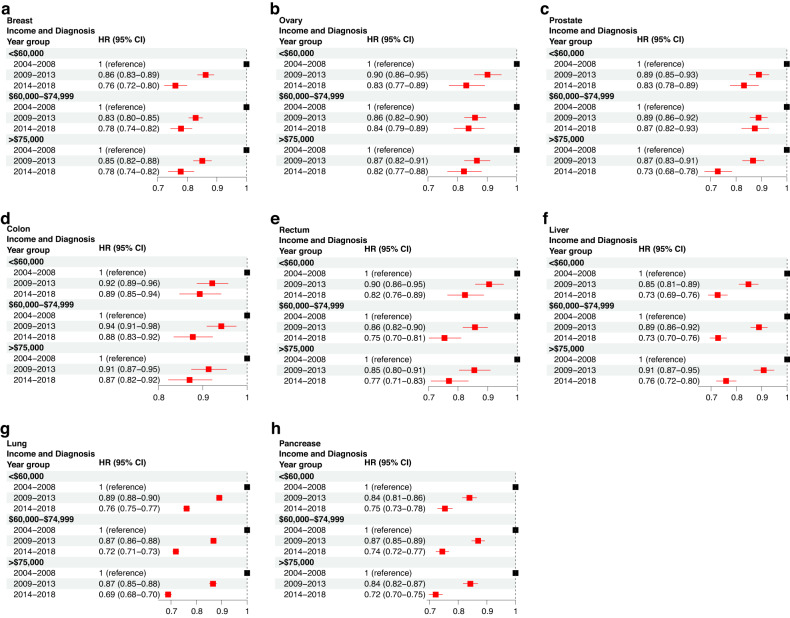
Multivariable Cox regression analysis to examine changes in CSS due to income levels between 2004 and 2018. The hazard ratio (HR) and 95% confidence interval (CI) is demonstrated for breast (**a**), ovary (**b**), prostate (**c**), colon (**d**), rectum (**e**), liver (**f**), lung (**g**), and pancreatic (**h**) cancer. The reference group for each comparison is denoted with an HR of 1 and the “reference” label.

In addition to age and race, we investigated the changes in socioeconomic disparity from 2004 to 2018. Our results indicated that in most cancer sites, except for breast and liver cancer, patients in the high-income group (>75 k) exhibited the greatest survival improvement, especially from 2014 to 2018 **(**Fig. 4). Of note, prostate cancer patients with a high income achieved a CSS increase by 27% (adjusted HR = 0.73, CI = [0.68–0.78]), compared with 17 and 13% achieved by the low- (adjusted HR = 0.83, CI = [0.78–0.89]) and intermediate-income (adjusted HR = 0.87, CI = [0.82 = 0.93]) patient groups. In breast and liver cancers, all income groups achieved a similar survival improvement across 2004–2018.

### Sex-based differences in cancer survival improvement from 2004–2018

**Fig. 5:**
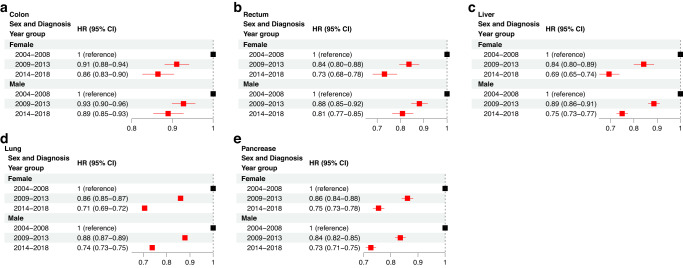
Multivariable Cox regression analysis to examine changes in CSS due to sex between 2004 and 2018. The hazard ratio (HR) and 95% confidence interval (CI) is demonstrated for colon (**a**), rectum (**b**), liver (**c**), lung (**d**), and pancreatic (**e**) cancer. The reference group for each comparison is denoted with an HR of 1 and the “reference” label.

We also examined changes CSS by sex from 2004 to 2018. In colon, rectal, liver, and lung cancers, female patients achieved greater improvements in CSS when compared to male patients, while in pancreatic cancer, male patients achieved greater survival improvements than female patients (Fig. 5).

### Disparity in prognosis improvement may be partially explained by earlier diagnosis

Finally, we compared the percentage of patients diagnosed at each cancer stage from 2009 to 2013 versus 2004–2008 and from 2014 to 2018 versus 2004–2008 (Table 1). In the latter comparison, black patients demonstrated an increase in stage I diagnosis across all eight cancer sites and a decrease in stage IV diagnosis in breast, rectal, liver, and pancreatic cancers. Across most racial groups and except for prostate, colon, and ovarian cancer, an increase in patients diagnosed at stage I and a decrease or stability in patients diagnosed at stage IV was seen. The trend is particularly clear in liver and pancreatic cancers, where all four groups experienced an increase in stage I diagnoses and a decrease in stage IV diagnoses comparing 2014–2018 to 2004–2008 data. In prostate cancer, a decrease was seen in stage II diagnoses, but an increase was seen in stage I, III, and IV diagnoses.

**Table 1:** Analysis comparing the percentage of patients diagnosed at each cancer stage from 2009 to 2013 versus 2004 to 2008 and from 2014 to 2018 versus 2004 to 2008 by cancer type and race.

		Y2009–2013 (percentage difference from Y2004-Y2008)				Y2014–2018 (percentage difference from Y2004–2008)			
Cancer Type	Race	Stage I	Stage II	Stage III	Stage IV	Stage I	Stage II	Stage III	Stage IV
Breast	White	0.016	−0.001	−0.017	0.002	0.094	−0.055	−0.041	0.002
	Asian	0.005	0.008	−0.016	0.002	0.074	−0.045	−0.034	0.006
	Black	0.035	−0.007	−0.023	−0.005	0.108	−0.048	−0.052	−0.008
	Hispanic	0.02	0.007	−0.029	0.002	0.094	−0.047	−0.051	0.005
Ovary	White	0.023	0.001	−0.005	−0.019	0.036	0.002	−0.034	−0.004
	Asian	0.03	0.01	−0.026	−0.014	−0.015	0.015	−0.011	0.01
	Black	0.007	−0.002	0.002	−0.008	0.019	−0.008	−0.036	0.026
	Hispanic	0.026	0.015	−0.02	−0.022	0.023	0.001	−0.035	0.01
Prostate	White	0.002	−0.046	0.021	0.022	0.159	−0.3	0.082	0.058
	Asian	0.002	−0.052	0.031	0.02	0.13	−0.303	0.106	0.067
	Black	0.001	−0.031	0.013	0.017	0.145	−0.261	0.068	0.048
	Hispanic	0.001	−0.05	0.019	0.03	0.16	−0.312	0.082	0.069
Colon	White	−0.002	−0.004	−0.001	0.007	−0.005	−0.005	0.002	0.008
	Asian	0.022	−0.032	0.001	0.01	0.012	−0.026	0.008	0.007
	Black	0.011	−0.016	−0.014	0.017	0.011	−0.02	−0.017	0.026
	Hispanic	−0.008	−0.001	−0.007	0.016	−0.014	−0.007	−0.002	0.024
Rectum	White	−0.044	0.001	0.034	0.009	−0.054	−0.033	0.068	0.018
	Asian	−0.002	0.007	0.008	−0.012	0.043	−0.035	0.023	−0.03
	Black	−0.039	−0.003	0.023	0.019	0.069	−0.063	0.016	−0.022
	Hispanic	−0.026	−0.021	0.06	−0.013	0.013	−0.046	0.063	−0.029
Liver	White	0.035	0.006	−0.002	−0.039	0.041	0.018	−0.033	−0.026
	Asian	0.03	−0.004	0.001	−0.028	0.056	0.001	−0.033	−0.023
	Black	0.031	0.021	−0.004	−0.048	0.028	0.024	−0.027	−0.025
	Hispanic	0.037	0.01	−0.008	−0.038	0.028	0.026	−0.026	−0.028
Lung	White	0.019	−0.004	−0.022	0.007	0.054	0.018	−0.059	−0.014
	Asian	0.027	−0.002	−0.022	−0.001	0.045	0.015	−0.088	0.029
	Black	0.018	−0.002	−0.022	0.005	0.048	0.021	−0.071	0.002
	Hispanic	0.024	−0.001	−0.019	−0.004	0.057	0.021	−0.055	−0.024
Pancreas	White	0.009	0.018	−0.007	−0.021	0.048	−0.033	0.011	−0.027
	Asian	0.019	0.028	−0.014	−0.034	0.066	−0.026	−0.011	−0.03
	Black	0.009	0.005	−0.006	−0.007	0.051	−0.028	0.016	−0.039
	Hispanic	0.015	0.014	0.001	−0.029	0.057	−0.035	0.014	−0.037

## Discussion

Using SEER data from 2004 to 2018, we investigated changes in racial, socioeconomic, age, and sex disparities in cancer survival for eight different cancer sites. Healthcare access and equity is an important issue in cancer research, and our present study demonstrated persistent racial differences in CSS for each of our eight studied cancer sites. For example, white patients have the highest CSS in breast, prostate, and rectal cancers while Asian patients have the highest CSS in ovarian, liver, lung, and pancreatic cancers. We found that black patients have significantly lower CSS in most cancer sites when compared to white patients after adjusting for age, cancer stage, and income, while Asian patients have significantly longer CSS in most cancer sites when compared to white patients, which is consistent with previous studies [16, 17]. One driver of these disparities may be due to differences in medical treatment and care for specific races despite efforts to equitize healthcare access. One study found that white patients were more likely to receive aggressive treatment for colorectal cancer [18]. Other studies have shown that African Americans with stage I/II non-small cell lung cancer are less likely to receive recommended treatment of surgery compared to white patients, even after controlling for income level and insurance status [19, 20]. Notably, while patients of all races experienced a significant improvement in CSS in 2009–2013 and 2014–2018 when compared to 2004–2008, black patients with breast, ovarian, and prostate cancer from 2014 to 2018 demonstrate lower survival than white patients from 2004 to 2008, despite experiencing improvements over time. Our study demonstrates that a clear and persistent racial difference in CSS is still seen, highlighting the need to improve care for specific patient populations.

Most cancers arise in patients over the age of 60, and due to advances in healthcare, the worldwide population is aging, with 20% of the world’s population estimated to be over the age of 60 by 2050 [21]. In our study, age disparities were noted for all cancer sites, with patients being diagnosed with cancer at an older age having a significantly worse CSS than patients diagnosed between the ages of 40 and 55. These results are consistent with a 2010 study of the SEER database, which found a lower CSS in all analyzed cancers in older patients, except in leukemia and Hodgkin lymphoma [22]. Older patients have higher rates of comorbidities, such as diabetes, high cholesterol, and hypertension, in addition to reduced immune function, making them susceptible to increased morbidity and mortality with concurrent cancer diagnoses. Additionally, molecular studies have demonstrated that the ageing microenvironment may play a role in the differential outcomes of young versus old patients in various forms of cancer [21]. Future basic and translational clinical research studies may benefit from more clearly outlining the differences between younger and older cancer patients to help improve therapy and outcomes in older patients, a group that our study has demonstrated suffers from lower CSS.

In addition to race and age, socioeconomic status, sex, and stage are additional important epidemiological and clinical factors that help us understand differences in CSS. In the present study, we found that patients with higher adjusted annual household incomes have higher CSS than patients with lower income levels, male patients have poorer CSS than female patients in most cancer types, and patients diagnosed at Stage IV have lower CSS than those diagnosed at an earlier stage. One study found that cancer death rates in men and women were 13 and 3% higher, respectively, in poorer counties compared to wealthy counties [23]. This trend may be partially explained by the fact that wealthier patients are significantly more likely to receive preventative cancer screening [24], and they may also have access to additional treatment modalities and care options [25]. This disparity highlights a need to improve access and affordability, increase preventative cancer screening in vulnerable populations, and reduce barriers to care [25]. Our results also suggest that socioeconomic disparities in CSS have grown wider between 2004 and 2018. For most cancer sites, except for breast and liver cancers, the highest income group achieved greater improvements in survival than the lower and middle income groups.

Finally, our present study also sought to characterize differences in survival improvement by race. From 2009 to 2018, for example, black patients have also seen significant survival improvements in each of the studied cancers. Part of this may be explained by increases in stage I diagnoses for black patients across all eight studied cancer sites and decreases in stage IV diagnoses for four cancer sites. While there still exist differences in overall survival, it appears that early diagnosis can help improve cancer mortality, especially in populations with lower overall survival. The present analysis also has several limitations, including the presence of unrecorded variables, variations in data coding and reporting, patient migration between SEER registry areas, missing data, and the potential for early censoring to indicate worse survival [26]. Another significant limitation is that the hospital-based data from SEER does not capture patient data from private clinics, outpatient radiation centers, nursing homes, or other outpatient physician offices, which are common treatment sites for certain cancers, such as breast and prostate cancer. Furthermore, certain patient populations may experience a higher rate of loss to follow-up and inflated survival. Additionally, the relationship between molecular receptors and race/ethnicity was not included in the present study due to data availability of molecularly defining receptors across all cancer sites, but analysis should be conducted in the future with datasets containing the necessary molecular data. Finally, income was calculated as an area-based measure, which is imperfect and can introduce inaccuracies, especially in large counties that may have a wide range of individual incomes.

In conclusion, analysis of the SEER data demonstrates persistent significant racial and socioeconomic disparities in cancer survival for all cancer sites. While racial disparities may have been reduced due to greater differential improvement of cancer survival in black patients when compared to patients of other races, black patients with breast, ovarian, and prostate cancer may remain at an increased risk. Additionally, socioeconomic disparities have widened as patients with a high adjusted household income achieved greater survival improvements than patients of low and middle incomes. Additionally, our present study provides an atlas to help view macro-level trends in cancer survival. The slower improvement in survival seen in certain cancers, such as prostate cancer, may be due to earlier advances that improved patient survival, while recent advances or trends, such as decreased smoking patterns in the US, have led to improved recent survival in other cancers (e.g., lung cancer). Taken together, our results are encouraging as improved survival was seen broadly across most cancer sites, and future studies may aim to focus on highly morbid cancers that have seen less improvement than other cancer sites over the years. Importantly, our study also indicates that more resources and interventions should be implemented to improve cancer treatment for specific patient groups with lower CSS.

## Supplementary information

Supplementary Materials

## Data Availability

Data is publicly available and accessible through the Surveillance, Epidemiology, and End Results (SEER) database (https://seer.cancer.gov/).
