# Supplementary material for: Racial and socioeconomic disparities in survival improvement of eight cancers
Source: BJC Rep. 2024 Mar 11;2:21. doi: 10.1038/s44276-024-00044-y (PMC11524065; doi:10.1038/s44276-024-00044-y)
Supplement: Supplementary file 1 — Supplementary Materials [file 44276_2024_44_MOESM1_ESM.docx]

**Racial and socioeconomic disparities in survival improvement of eight cancers**

Vikram Shaw, MS^1^, Baoyi Zhang, PhD^1^, Emily Tang, BS^2^, William Peng^3^, Christopher Amos, PhD^1,4,5*^, Chao Cheng, PhD^1,4*^

1. Institute for Clinical and Translational Research, Baylor College of Medicine, Houston, TX 77030, United States.

2. Rice University, Houston, TX 77005, United States.

3. University of Houston, Houston TX 77004, United States.

4. Section of Epidemiology and Population Sciences, Department of Medicine, Baylor College of Medicine, Houston, TX 77030, United States.

5. Dan L Duncan Comprehensive Cancer Center, Baylor College of Medicine, Houston, TX 77030, United States.

**
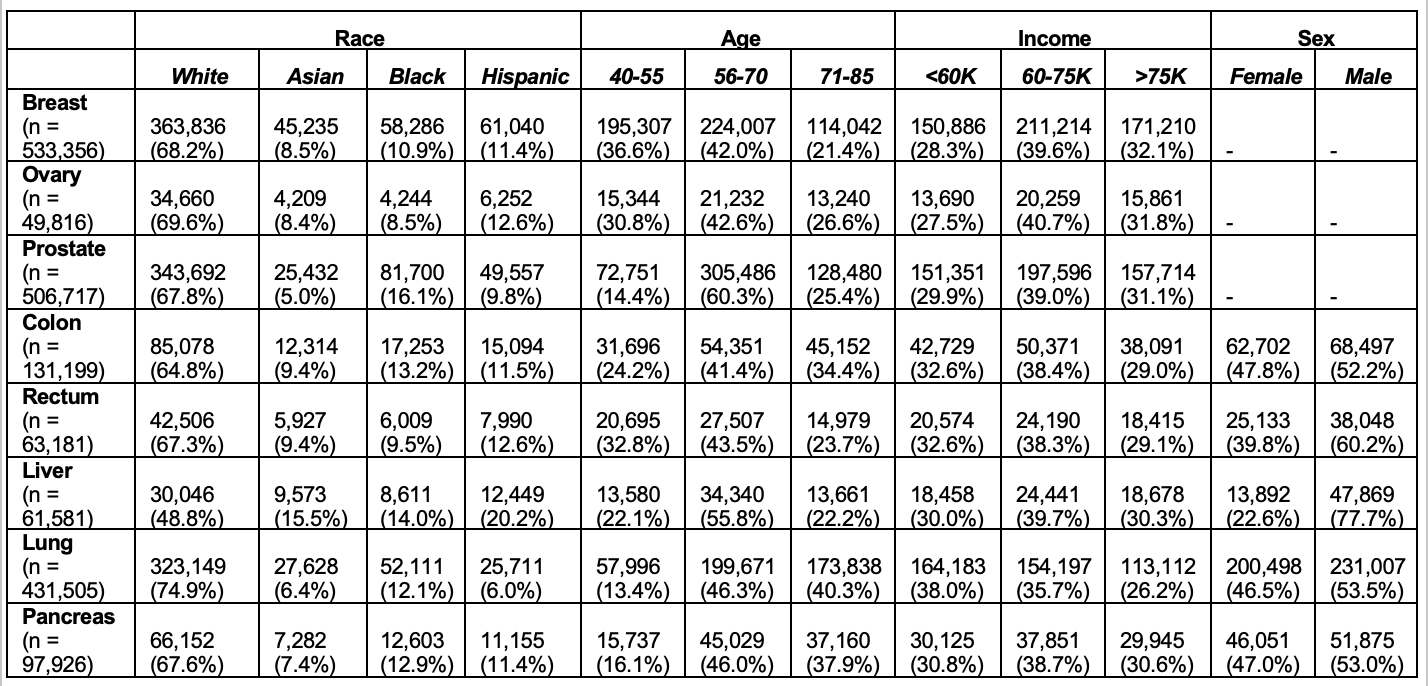
**

**Supplementary Table 1.** Descriptive statistics for each of the eight studied cancers, including patient race, age, income, and sex.


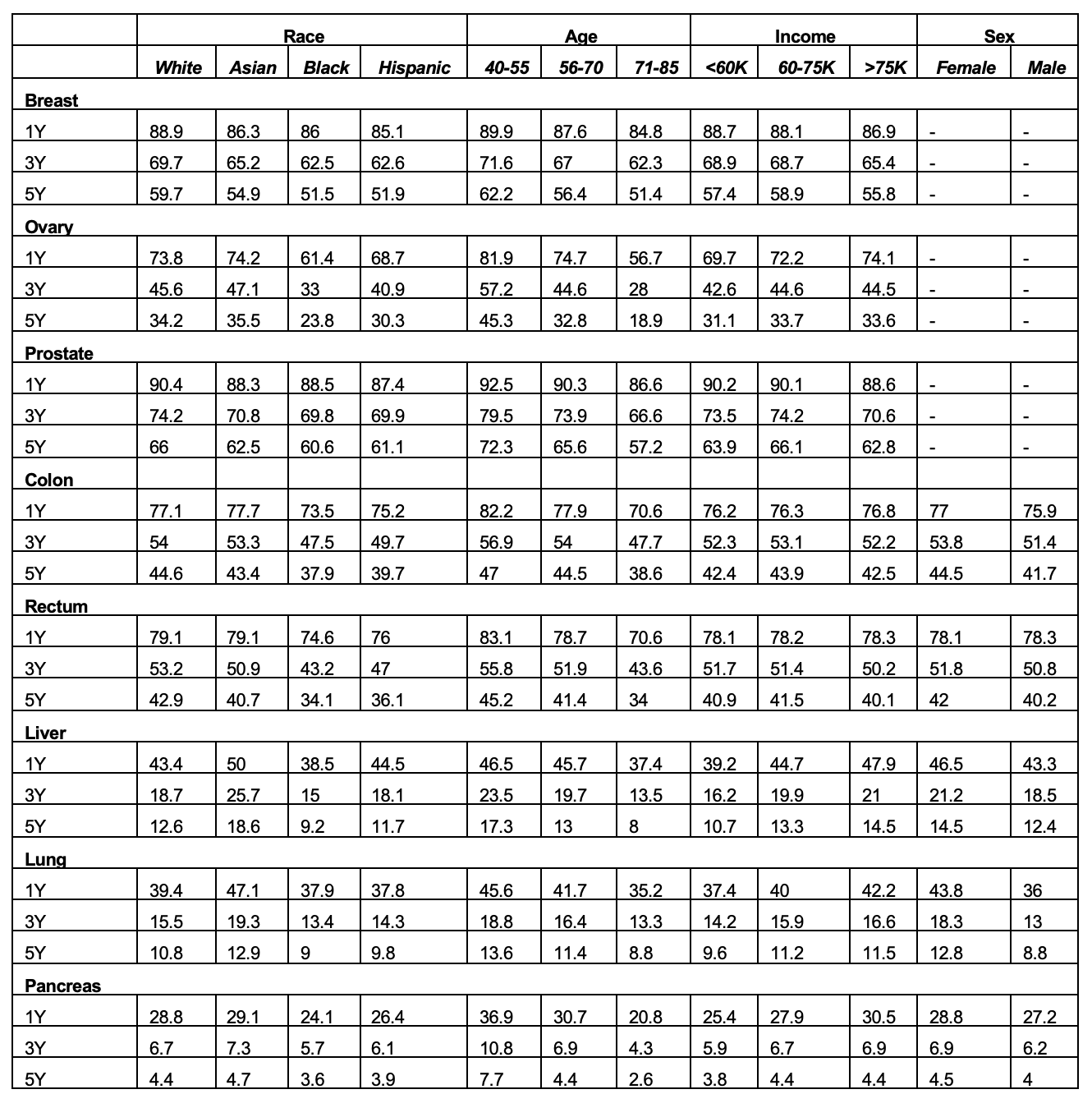


**Supplementary Table 2.** Cancer-specific survival (CSS) as a percentage of population after 1, 3, and 5 years for each cancer type, stratified by race, age, income, and sex.
